# (3-Pyrid­yl)methanaminium 4-nitro­phenolate 4-nitro­phenol solvate

**DOI:** 10.1107/S1600536810021902

**Published:** 2010-06-16

**Authors:** Yuan Zhang, Meng Ting Han

**Affiliations:** a Ordered Matter Science Research Center, College of Chemistry and Chemical, Engineering, Southeast University, Nanjing 211189, People’s Republic of China

## Abstract

In the crystal structure of the title compound, C_6_H_9_N_2_^+^·C_6_H_4_NO_3_^−^·C_6_H_5_NO_3_, ions and mol­ecules are connected *via* inter­molecular N—H⋯O, N—H⋯N, O—H⋯O and C—H⋯O hydrogen bonds into a three-dimensional network.

## Related literature

For background to the development of ferroelectric pure organic or inorganic compounds, see: Haertling *et al.* (1999 ▶); Homes *et al.* (2001 ▶). For our recent reports on the synthesis of a variety of compounds which have potential piezoelectric and ferroelectric properties, see: Fu *et al.* (2009 ▶); Hang *et al.* (2009 ▶).
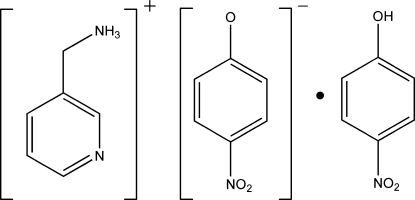


## Experimental

### 

#### Crystal data

C_6_H_9_N_2_^+^·C_6_H_4_NO_3_^−^·C_6_H_5_NO_3_
*M* *_r_* = 386.36
Triclinic, 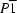

*a* = 6.3666 (13) Å
*b* = 7.4451 (15) Å
*c* = 21.262 (4) Å
α = 92.41 (3)°
β = 95.56 (3)°
γ = 113.99 (3)°
*V* = 912.8 (3) Å^3^
*Z* = 2
Mo *K*α radiation
μ = 0.11 mm^−1^
*T* = 293 K
0.20 × 0.20 × 0.20 mm

#### Data collection

Rigaku Mercury2 diffractometer
Absorption correction: multi-scan (*CrystalClear*; Rigaku, 2005 ▶) *T*_min_ = 0.825, *T*_max_ = 1.000
9547 measured reflections
4182 independent reflections
2896 reflections with *I* > 2σ(*I*)
*R*_int_ = 0.034

#### Refinement

*R*[*F* ^2^ > 2σ(*F* ^2^)] = 0.054
*wR*(*F* ^2^) = 0.140
*S* = 1.05
4182 reflections
253 parameters
H-atom parameters constrained
Δρ_max_ = 0.16 e Å^−3^
Δρ_min_ = −0.25 e Å^−3^

### 

Data collection: *CrystalClear* (Rigaku, 2005 ▶); cell refinement: *CrystalClear*; data reduction: *CrystalClear*; program(s) used to solve structure: *SHELXS97* (Sheldrick, 2008 ▶); program(s) used to refine structure: *SHELXL97* (Sheldrick, 2008 ▶); molecular graphics: *SHELXTL* (Sheldrick, 2008 ▶); software used to prepare material for publication: *SHELXTL*.

## Supplementary Material

Crystal structure: contains datablocks I, global. DOI: 10.1107/S1600536810021902/jh2162sup1.cif

Structure factors: contains datablocks I. DOI: 10.1107/S1600536810021902/jh2162Isup2.hkl

Additional supplementary materials:  crystallographic information; 3D view; checkCIF report

## Figures and Tables

**Table 1:** Hydrogen-bond geometry (Å, °)

*D*—H⋯*A*	*D*—H	H⋯*A*	*D*⋯*A*	*D*—H⋯*A*
N1—H1*A*⋯O2^i^	0.89	2.09	2.952 (2)	162
N1—H1*B*⋯O3^ii^	0.89	1.87	2.753 (2)	169
N1—H1*C*⋯N2^iii^	0.89	2.16	2.866 (2)	136
O4—H4*A*⋯O3	0.96	1.58	2.5385 (19)	173
C1—H1*D*⋯O5^iv^	0.93	2.52	3.229 (3)	133
C2—H2*A*⋯O6^v^	0.93	2.58	3.327 (3)	138
C8—H10*A*⋯O4^iii^	0.93	2.54	3.462 (3)	169

Symmetry codes: (i) 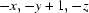; (ii) 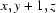; (iii) 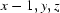; (iv) 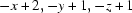; (v) 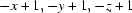.

**Fig. 1.**
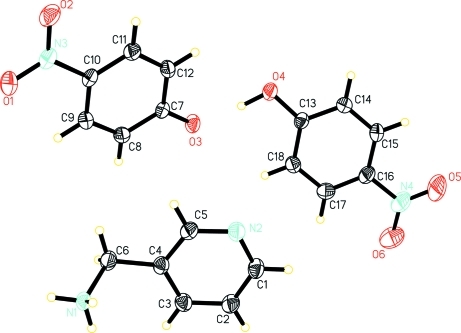
Perspective structure of the title compound, showing the atom-numbering scheme. Displacement ellipsoids are drawn at the 30% probability level.

**Fig. 2.**
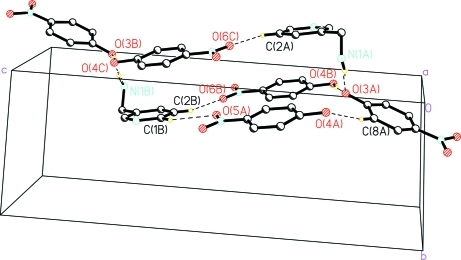
The crystal packing of the title compound viewed along the a axis showing the hydrogen bondings network. Some of the H atoms have been ommitted for clarity.

## supplementary crystallographic information

### Comment

At present, much attention in ferroelectric material field is focused on developing ferroelectric pure organic or inorganic compounds (Haertling *et al.* 1999; Homes *et al.* 2001). Recently we have reported the synthesis of a variety of compounds (Fu *et al.*, 2009; Hang *et al.*, 2009), which have potential piezoelectric and ferroelectric properties. In order to find more dielectric ferroelectric materials, we investigate the physical properties of the title compound(Fig. 1). The dielectric constant of the title compound as a function of temperature indicates that the permittivity is basically temperature-independent (dielectric constant equaling to 2.8 to 4.6), suggesting that this compound should be not a real ferroelectrics or there may be no distinct phase transition occurred within the measured temperature range. Similarly, below the melting point (399 K) of the compound, the dielectric constant as a function of temperature also goes smoothly, and there is no dielectric anomaly observed (dielectric constant equaling to 2.8 to 4.6).Herein, we report the synthesis and crystal structure of the title compound.

The molecular structure of the title compound is shown in Fig. 1. There are one 4-nitrophenolate anion, an substituted ammonium cation and a neutral 4-nitrophenol molecule in the asymmetric unit. Molecules of the title compound have normal geometric parameters. The bond lengths and angles are within their normal ranges. All pyridine rings are, of course, planar. As can be seen from the packing diagram (Fig. 2), molecules are connected *via* intermolecular C—H···O and O—H···N hydrogen bonds to form a three dimensional network. Dipole–dipole and van der Waals interactions are effective in the molecular packing.

### Experimental

4-nitrophenol (2.085 g, 0.015 mol) was added slowly to a solution of pyridin-3-ylmethanamine (1.62 g, 0.015 mol) in methanol.After several days, the title compound was formed and recrystallized from solution to afford colourless prismatic crystals suitable for X-ray analysis.

### Refinement

H atoms were positioned geometrically and refined using a riding model, with C—H(aromatic) = 0.93 and 0.97 (methylene) Å, N—H = 0.89 Å and O—H = 0.97 Å and with *U*_iso_(H) = 1.3–1.5*U*_eq_(C, N, O).

### Crystal data

**Table:**

C_6_H_9_N_2_^+^·C_6_H_4_NO_3_^−^·C_6_H_5_nO_3_	*Z* = 2
*M**_r_* = 386.36	*F*(000) = 404
Triclinic, *P*1	*D*_x_ = 1.406 Mg m^−^^3^
Hall symbol: -P 1	Mo *K*α radiation, λ = 0.71073 Å
*a* = 6.3666 (13) Å	Cell parameters from 4182 reflections
*b* = 7.4451 (15) Å	θ = 2.6–27.5°
*c* = 21.262 (4) Å	µ = 0.11 mm^−^^1^
α = 92.41 (3)°	*T* = 293 K
β = 95.56 (3)°	Prism, colorless
γ = 113.99 (3)°	0.20 × 0.20 × 0.20 mm
*V* = 912.8 (3) Å^3^	

### Data collection

**Table:**

Rigaku Mercury2 diffractometer	4182 independent reflections
Radiation source: fine-focus sealed tube	2896 reflections with *I* > 2σ(*I*)
graphite	*R*_int_ = 0.034
Detector resolution: 13.6612 pixels mm^-1^	θ_max_ = 27.5°, θ_min_ = 3.0°
CCD_Profile_fitting scans	*h* = −8→8
Absorption correction: multi-scan (*CrystalClear*; Rigaku, 2005)	*k* = −9→9
*T*_min_ = 0.825, *T*_max_ = 1.000	*l* = −27→27
9547 measured reflections	

### Refinement

**Table:**

Refinement on *F*^2^	Primary atom site location: structure-invariant direct methods
Least-squares matrix: full	Secondary atom site location: difference Fourier map
*R*[*F*^2^ > 2σ(*F*^2^)] = 0.054	Hydrogen site location: inferred from neighbouring sites
*wR*(*F*^2^) = 0.140	H-atom parameters constrained
*S* = 1.05	*w* = 1/[σ^2^(*F*_o_^2^) + (0.0576*P*)^2^ + 0.2125*P*] where *P* = (*F*_o_^2^ + 2*F*_c_^2^)/3
4182 reflections	(Δ/σ)_max_ < 0.001
253 parameters	Δρ_max_ = 0.16 e Å^−^^3^
0 restraints	Δρ_min_ = −0.25 e Å^−^^3^

### Special details

**Table:**

Geometry. All e.s.d.'s (except the e.s.d. in the dihedral angle between two l.s. planes) are estimated using the full covariance matrix. The cell e.s.d.'s are taken into account individually in the estimation of e.s.d.'s in distances, angles and torsion angles; correlations between e.s.d.'s in cell parameters are only used when they are defined by crystal symmetry. An approximate (isotropic) treatment of cell e.s.d.'s is used for estimating e.s.d.'s involving l.s. planes.
Refinement. Refinement of *F*^2^ against ALL reflections. The weighted *R*-factor *wR* and goodness of fit *S* are based on *F*^2^, conventional *R*-factors *R* are based on *F*, with *F* set to zero for negative *F*^2^. The threshold expression of *F*^2^ > σ(*F*^2^) is used only for calculating *R*-factors(gt) *etc*. and is not relevant to the choice of reflections for refinement. *R*-factors based on *F*^2^ are statistically about twice as large as those based on *F*, and *R*- factors based on ALL data will be even larger.

### Fractional atomic coordinates and isotropic or equivalent isotropic displacement parameters (Å^2^)

**Table:**

	*x*	*y*	*z*	*U*_iso_*/*U*_eq_	
O3	0.3538 (2)	0.0490 (2)	0.19323 (6)	0.0471 (3)	
O4	0.7520 (2)	0.0641 (2)	0.23068 (6)	0.0490 (4)	
H4A	0.6012	0.0634	0.2194	0.074*	
C10	0.2665 (3)	0.2431 (3)	0.01944 (8)	0.0389 (4)	
N3	0.2243 (3)	0.2980 (2)	−0.04283 (8)	0.0481 (4)	
C7	0.3307 (3)	0.1152 (3)	0.13814 (8)	0.0369 (4)	
O2	0.3528 (3)	0.2970 (2)	−0.08282 (7)	0.0609 (4)	
O1	0.0584 (3)	0.3409 (3)	−0.05577 (7)	0.0687 (5)	
C9	0.1125 (3)	0.2297 (3)	0.06264 (9)	0.0442 (5)	
H9A	−0.0114	0.2633	0.0522	0.053*	
C8	0.1442 (3)	0.1664 (3)	0.12100 (9)	0.0438 (4)	
H10A	0.0403	0.1572	0.1499	0.053*	
C13	0.8177 (3)	0.1128 (3)	0.29326 (8)	0.0388 (4)	
C12	0.4868 (3)	0.1361 (3)	0.09345 (9)	0.0445 (5)	
H12A	0.6144	0.1074	0.1040	0.053*	
C11	0.4552 (3)	0.1978 (3)	0.03479 (9)	0.0445 (5)	
H11A	0.5589	0.2091	0.0057	0.053*	
C18	0.6884 (3)	0.1667 (3)	0.33310 (9)	0.0488 (5)	
H18A	0.5513	0.1731	0.3165	0.059*	
C16	0.9669 (4)	0.2016 (3)	0.42097 (9)	0.0523 (5)	
C14	1.0262 (3)	0.1105 (3)	0.31834 (9)	0.0476 (5)	
H14A	1.1167	0.0798	0.2917	0.057*	
C15	1.0994 (4)	0.1534 (3)	0.38222 (10)	0.0530 (5)	
H15A	1.2379	0.1497	0.3991	0.064*	
C17	0.7627 (4)	0.2106 (3)	0.39719 (10)	0.0570 (6)	
H17A	0.6761	0.2459	0.4240	0.068*	
O5	1.2363 (4)	0.2523 (5)	0.50752 (9)	0.1289 (10)	
O6	0.9263 (4)	0.2807 (4)	0.52407 (9)	0.1112 (8)	
N4	1.0492 (5)	0.2488 (4)	0.48875 (10)	0.0792 (7)	
N1	−0.0537 (3)	0.7260 (2)	0.20070 (7)	0.0414 (4)	
H1A	−0.1686	0.7006	0.1696	0.062*	
H1B	0.0690	0.8325	0.1935	0.062*	
H1C	−0.0989	0.7476	0.2375	0.062*	
N2	0.5902 (3)	0.6209 (3)	0.28249 (8)	0.0502 (4)	
C1	0.5678 (4)	0.6498 (3)	0.34345 (9)	0.0485 (5)	
H1D	0.6919	0.6693	0.3739	0.058*	
C4	0.2013 (3)	0.5896 (3)	0.25514 (9)	0.0398 (4)	
C5	0.4074 (3)	0.5913 (3)	0.24015 (9)	0.0466 (5)	
H5A	0.4205	0.5703	0.1975	0.056*	
C3	0.1849 (3)	0.6229 (3)	0.31859 (9)	0.0469 (5)	
H3A	0.0499	0.6254	0.3309	0.056*	
C6	0.0087 (4)	0.5549 (3)	0.20322 (10)	0.0491 (5)	
H6A	0.0559	0.5305	0.1628	0.059*	
H6C	−0.1262	0.4384	0.2101	0.059*	
C2	0.3695 (3)	0.6522 (3)	0.36331 (9)	0.0490 (5)	
H2A	0.3605	0.6733	0.4062	0.059*	

### Atomic displacement parameters (Å^2^)

**Table:**

	*U*^11^	*U*^22^	*U*^33^	*U*^12^	*U*^13^	*U*^23^
O3	0.0441 (8)	0.0624 (9)	0.0358 (7)	0.0228 (7)	0.0022 (6)	0.0128 (6)
O4	0.0430 (8)	0.0695 (9)	0.0332 (7)	0.0231 (7)	−0.0002 (6)	0.0017 (6)
C10	0.0433 (10)	0.0396 (10)	0.0295 (9)	0.0146 (8)	−0.0031 (7)	0.0008 (7)
N3	0.0561 (11)	0.0485 (10)	0.0345 (9)	0.0185 (8)	−0.0034 (8)	0.0026 (7)
C7	0.0358 (9)	0.0402 (10)	0.0305 (9)	0.0128 (8)	−0.0018 (7)	0.0015 (7)
O2	0.0672 (10)	0.0792 (11)	0.0352 (8)	0.0280 (8)	0.0083 (7)	0.0124 (7)
O1	0.0807 (12)	0.0936 (12)	0.0480 (9)	0.0546 (10)	−0.0054 (8)	0.0147 (8)
C9	0.0437 (11)	0.0536 (11)	0.0404 (10)	0.0265 (9)	−0.0004 (8)	0.0038 (9)
C8	0.0433 (11)	0.0561 (12)	0.0370 (10)	0.0249 (9)	0.0074 (8)	0.0044 (9)
C13	0.0389 (10)	0.0422 (10)	0.0324 (9)	0.0143 (8)	0.0020 (7)	0.0050 (8)
C12	0.0398 (10)	0.0605 (12)	0.0386 (10)	0.0267 (9)	0.0014 (8)	0.0068 (9)
C11	0.0412 (10)	0.0563 (12)	0.0360 (10)	0.0198 (9)	0.0059 (8)	0.0037 (8)
C18	0.0410 (11)	0.0651 (13)	0.0432 (11)	0.0253 (10)	0.0035 (8)	0.0031 (9)
C16	0.0573 (13)	0.0654 (13)	0.0322 (10)	0.0250 (11)	−0.0012 (9)	0.0009 (9)
C14	0.0441 (11)	0.0602 (12)	0.0420 (11)	0.0264 (10)	0.0024 (8)	−0.0029 (9)
C15	0.0458 (12)	0.0689 (14)	0.0451 (12)	0.0283 (11)	−0.0090 (9)	−0.0022 (10)
C17	0.0527 (13)	0.0778 (15)	0.0436 (12)	0.0291 (12)	0.0128 (9)	−0.0005 (11)
O5	0.1125 (19)	0.228 (3)	0.0508 (12)	0.088 (2)	−0.0332 (12)	−0.0189 (14)
O6	0.1272 (19)	0.172 (2)	0.0420 (10)	0.0706 (18)	0.0152 (11)	−0.0083 (12)
N4	0.0898 (17)	0.1082 (18)	0.0373 (11)	0.0416 (14)	−0.0021 (11)	−0.0018 (11)
N1	0.0396 (9)	0.0564 (10)	0.0309 (8)	0.0226 (8)	0.0024 (6)	0.0071 (7)
N2	0.0444 (10)	0.0676 (11)	0.0412 (9)	0.0260 (9)	0.0038 (7)	0.0050 (8)
C1	0.0471 (11)	0.0589 (12)	0.0383 (11)	0.0217 (10)	−0.0015 (8)	0.0085 (9)
C4	0.0421 (10)	0.0377 (10)	0.0379 (10)	0.0159 (8)	−0.0004 (8)	0.0047 (8)
C5	0.0499 (12)	0.0566 (12)	0.0343 (10)	0.0232 (10)	0.0043 (8)	0.0016 (9)
C3	0.0431 (11)	0.0576 (12)	0.0430 (11)	0.0232 (10)	0.0075 (8)	0.0076 (9)
C6	0.0529 (12)	0.0454 (11)	0.0462 (12)	0.0212 (10)	−0.0091 (9)	−0.0012 (9)
C2	0.0507 (12)	0.0643 (13)	0.0329 (10)	0.0235 (10)	0.0088 (8)	0.0081 (9)

### Geometric parameters (Å, °)

**Table:**

O3—C7	1.308 (2)	C14—C15	1.373 (3)
O4—C13	1.344 (2)	C14—H14A	0.9300
O4—H4A	0.9646	C15—H15A	0.9300
C10—C9	1.385 (3)	C17—H17A	0.9300
C10—C11	1.387 (3)	O5—N4	1.209 (3)
C10—N3	1.435 (2)	O6—N4	1.218 (3)
N3—O1	1.232 (2)	N1—C6	1.482 (2)
N3—O2	1.238 (2)	N1—H1A	0.8900
C7—C8	1.409 (3)	N1—H1B	0.8900
C7—C12	1.409 (3)	N1—H1C	0.8900
C9—C8	1.371 (3)	N2—C1	1.336 (3)
C9—H9A	0.9300	N2—C5	1.336 (3)
C8—H10A	0.9300	C1—C2	1.376 (3)
C13—C14	1.389 (3)	C1—H1D	0.9300
C13—C18	1.389 (3)	C4—C5	1.375 (3)
C12—C11	1.372 (3)	C4—C3	1.383 (3)
C12—H12A	0.9300	C4—C6	1.498 (3)
C11—H11A	0.9300	C5—H5A	0.9300
C18—C17	1.378 (3)	C3—C2	1.375 (3)
C18—H18A	0.9300	C3—H3A	0.9300
C16—C15	1.370 (3)	C6—H6A	0.9700
C16—C17	1.377 (3)	C6—H6C	0.9700
C16—N4	1.462 (3)	C2—H2A	0.9300
			
C13—O4—H4A	110.0	C14—C15—H15A	120.3
C9—C10—C11	121.06 (17)	C16—C17—C18	119.14 (19)
C9—C10—N3	119.04 (17)	C16—C17—H17A	120.4
C11—C10—N3	119.86 (18)	C18—C17—H17A	120.4
O1—N3—O2	121.47 (17)	O5—N4—O6	122.7 (2)
O1—N3—C10	119.44 (17)	O5—N4—C16	118.4 (2)
O2—N3—C10	119.07 (17)	O6—N4—C16	119.0 (2)
O3—C7—C8	120.54 (17)	C6—N1—H1A	109.5
O3—C7—C12	122.14 (16)	C6—N1—H1B	109.5
C8—C7—C12	117.31 (16)	H1A—N1—H1B	109.5
C8—C9—C10	119.43 (17)	C6—N1—H1C	109.5
C8—C9—H9A	120.3	H1A—N1—H1C	109.5
C10—C9—H9A	120.3	H1B—N1—H1C	109.5
C9—C8—C7	121.42 (18)	C1—N2—C5	116.81 (18)
C9—C8—H10A	119.3	N2—C1—C2	123.07 (19)
C7—C8—H10A	119.3	N2—C1—H1D	118.5
O4—C13—C14	117.63 (17)	C2—C1—H1D	118.5
O4—C13—C18	123.06 (17)	C5—C4—C3	117.13 (18)
C14—C13—C18	119.31 (17)	C5—C4—C6	119.56 (18)
C11—C12—C7	121.54 (17)	C3—C4—C6	123.31 (18)
C11—C12—H12A	119.2	N2—C5—C4	124.60 (18)
C7—C12—H12A	119.2	N2—C5—H5A	117.7
C12—C11—C10	119.20 (18)	C4—C5—H5A	117.7
C12—C11—H11A	120.4	C2—C3—C4	119.60 (19)
C10—C11—H11A	120.4	C2—C3—H3A	120.2
C17—C18—C13	120.22 (19)	C4—C3—H3A	120.2
C17—C18—H18A	119.9	N1—C6—C4	111.67 (16)
C13—C18—H18A	119.9	N1—C6—H6A	109.3
C15—C16—C17	121.48 (19)	C4—C6—H6A	109.3
C15—C16—N4	118.6 (2)	N1—C6—H6C	109.3
C17—C16—N4	119.9 (2)	C4—C6—H6C	109.3
C15—C14—C13	120.36 (19)	H6A—C6—H6C	107.9
C15—C14—H14A	119.8	C3—C2—C1	118.79 (19)
C13—C14—H14A	119.8	C3—C2—H2A	120.6
C16—C15—C14	119.43 (19)	C1—C2—H2A	120.6
C16—C15—H15A	120.3		

### Hydrogen-bond geometry (Å, °)

**Table:**

*D*—H···*A*	*D*—H	H···*A*	*D*···*A*	*D*—H···*A*
N1—H1A···O2^i^	0.89	2.09	2.952 (2)	162
N1—H1B···O3^ii^	0.89	1.87	2.753 (2)	169
N1—H1C···N2^iii^	0.89	2.16	2.866 (2)	136
O4—H4A···O3	0.96	1.58	2.5385 (19)	173
C1—H1D···O5^iv^	0.93	2.52	3.229 (3)	133
C2—H2A···O6^v^	0.93	2.58	3.327 (3)	138
C8—H10A···O4^iii^	0.93	2.54	3.462 (3)	169

Symmetry codes: (i) −*x*, −*y*+1, −*z*; (ii) *x*, *y*+1, *z*; (iii) *x*−1, *y*, *z*; (iv) −*x*+2, −*y*+1, −*z*+1; (v) −*x*+1, −*y*+1, −*z*+1.
